# Different Assembly Processes Drive Shifts in Species and Functional Composition in Experimental Grasslands Varying in Sown Diversity and Community History

**DOI:** 10.1371/journal.pone.0101928

**Published:** 2014-07-16

**Authors:** Christiane Roscher, Jens Schumacher, Uta Gerighausen, Bernhard Schmid

**Affiliations:** 1 Department of Community Ecology, Helmholtz Centre for Environmental Research, UFZ, Halle, Germany; 2 Institute of Stochastics, Friedrich Schiller University Jena, Germany; 3 Max Planck Institute for Biogeochemistry, Jena, Germany; 4 Institute of Evolutionary Biology and Environmental Studies and Zurich-Basel Plant Science Center, University of Zurich, Zurich, Switzerland; University of Northampton, United Kingdom

## Abstract

**Background:**

The prevalence of different biotic processes (limiting similarity, weaker competitor exclusion) and historical contingency due to priority effects are in the focus of ongoing discussions about community assembly and non-random functional trait distributions.

**Methodology/Principal Findings:**

We experimentally manipulated assembly history in a grassland biodiversity experiment (Jena Experiment) by applying two factorially crossed split-plot treatments to all communities: (i) duration of weeding (never weeded since sowing or cessation of weeding after 3 or 6 years); (ii) seed addition (control vs. seed addition 4 years after sowing). Spontaneous colonization of new species in the control treatment without seed addition increased realized species richness and functional richness (FRic), indicating continuously denser packing of niches. Seed addition resulted in forced colonization and increased realized species richness, FRic, functional evenness (FEve) and functional divergence (FDiv), i.e. higher abundances of species with extreme trait values. Furthermore, the colonization of new species led to a decline in FEve through time, suggesting that weaker competitors were reduced in abundance or excluded. Communities with higher initial species richness or with longer time since cessation of weeding were more restricted in the entry of new species and showed smaller increases in FRic after seed addition than other communities. The two assembly-history treatments caused a divergence of species compositions within communities originally established with the same species. Communities originally established with different species converged in species richness and functional trait composition over time, but remained more distinct in species composition.

**Conclusions/Significance:**

Contrasting biotic processes (limiting similarity, weaker competitor exclusion) increase functional convergence between communities initially established with different species. Historical contingency with regard to realized species compositions could not be eradicated by cessation of weeding or forced colonization and was still detectable 5 years after application of these treatments, providing evidence for the role of priority effects in community assembly.

## Introduction

A deeper insight into the mechanisms which control community assembly is central to understand ecosystem functioning and the maintenance of biodiversity. Functional diversity, i.e. the extent of trait differences among co-occurring species [1], has been proposed as an important characteristic of biological assemblages. The intimate link between traits and the functioning of organisms [2] suggests that patterns of functional trait distributions within and between communities may provide insights into the operation of non-neutral community assembly rules [3], [4]. Given a regional species pool with unlimited dispersal, community assembly is often assumed to result from two distinct non-random processes of species sorting: environmental filtering (abiotic filters) and species interactions (biotic filters). Environmental filtering is likely to reduce functional diversity by selecting species with similar ecological characteristics ( = trait convergence) which are able to tolerate the local environment [5], [6]. Biotic processes may produce different patterns of trait distribution. Niche differentiation through resource partitioning prevents coexisting species from being too similar (i.e. limiting similarity [7], [8]) and is expected to select for species with different ecological characteristics ( = trait divergence). However, it also has been discussed that competition may increase similarity among species ( = trait convergence), when species with similar traits compete relatively equally, while excluding weaker competitors with different traits (i.e. equalizing fitness processes [8], [9]). This niche-based view of community assembly has been challenged by the “neutral theory” [10] assuming that functional differences do not play a role in community assembly and that the fitness of all species in a community is equivalent. Different community histories, i.e. priority effects due to differences in the initial floristic composition, the order of arrival of new species and their initial establishment, may also impact the outcome of community assembly and prevent convergence in species and functional trait composition under uniform environmental conditions [11]–[13].

In natural ecosystems, the importance of biotic processes causing trait convergence and divergence in community assembly is difficult to disentangle because abiotic processes may also cause trait convergence or counterbalance biotic trait divergence [14], [15]. In addition, long-term effects due to priority effects and the role of dispersal limitation are hard to identify in communities with unknown assembly history. Biodiversity experiments, where abiotic effects are controlled for by randomizing plots with different plant diversity under relatively homogeneous environmental conditions are an opportunity to study biotic processes of community assembly [16]. In previous experiments it has been shown that, when experimental communities of different species richness are opened to colonization of new species, realized species richness can converge rapidly to similar levels [17]. Convergence can be reached even faster when dispersal limitation is reduced by seed addition [18]. However, convergence of species richness may not be paralleled by convergence of functional trait distribution or species composition and thus provides only limited insight into community assembly. Community-wide use of available niche space encompasses the following components: (i) the amount of niche space spanned out by species in a community (functional richness; FRic), (ii) the evenness of abundance distribution of functionally different species (functional evenness; FEve), and (iii) the degree to which the abundance distribution of functionally different species maximizes divergence in functional trait distributions within a community (functional divergence; FDiv) [19].

In the present study, we used experimental plots of a grassland biodiversity experiment (Jena Experiment [20]) initially established with different species richness (1, 2, 4, 8, and 16 species) and functional group number and composition (1 to 4) to study community assembly processes in response to two factorially crossed split-plot treatments [21]: (i) duration of weeding leading to different colonization periods of communities (cessation of weeding 6 years after sowing < cessation of weeding 3 years after sowing < never weeded since sowing); (ii) seed addition (control vs. seed addition 4 years after establishment of initial species compositions). During a 5-year study period following the seed addition we monitored species richness and composition and different components of functional diversity (FRic, FEve, FDiv [22]). We assume that our experimental approach controls for abiotic processes of community assembly and that seed addition removes dispersal limitation. We test the following hypothesis: (1) functional richness (FRic) increases in parallel to realized species richness during colonization due to limiting similarity; (2) increasing functional richness is succeeded by a decline in functional evenness (FEve) and functional divergence (FDiv) due to weaker competitor exclusion; (3) the removal of dispersal limitation due to seed addition increases the chance for the establishment of more species with traits advantageous in competition and weaker competitor exclusion, thus reducing realized species richness and FRic in the longer term; (4) historical contingency, i.e. priority effects due to initial species diversity and longer colonization periods, decelerate species gain and increase in FRic; (5) contrasting biotic processes during community assembly increase similarity in functional trait composition between communities through time, but historical contingency remains visible in distinct realized species compositions of communities initially established with different species richness and composition.

## Materials and Methods

### Study site and experimental design

The study was conducted on plots of the Jena Experiment, a large grassland biodiversity experiment established in 2002 on former agricultural land [20]. The experimental site is situated in the floodplain of the river Saale north to the city of Jena (Thuringia, Germany, 50°55′ N, 11°35′ E, 130 m a.s.l.), where a Eutric Fluvisol developed from up to 2 m thick fluvial sediments. A gradient in soil texture ranging from sandy loam in the vicinity of the river to silty clay with increasing distance from the river is due to fluvial dynamics. Mean annual air temperature is 9.3 °C and mean annual precipitation is 587 mm in the area around Jena [23]. The field site was rented by the research consortium of the Jena Experiment from an agricultural collective for the duration of the research grant. No specific permission was required for the described field study. The Jena Experiment field is not subject to protection for nature conservation and the study did not involve endangered or protected species.

The species pool of the experiment consisted of 60 Central European plant species common in semi-natural grasslands (Arrhenatherion community [24]). The species were categorized into four functional groups: grasses (16 species), legumes (12 species), tall herbs (20 species), and small herbs (12 species). The Jena Experiment comprises 78 plots of 20×20 m size. The design combines the factors species richness (1, 2, 4, 8, and 16) and functional group number (1, 2, 3, and 4) in a close-to-orthogonal way with the restriction that plant functional group number cannot exceed species number in a given mixture. Each species-richness level had 16 replicates, except for 14 replicates of the 16-species mixtures because pure legume and small herb mixtures with 16 species were not possible [20]. Mixture compositions were determined by random drawing with replacement. Plots were assigned to four experimental blocks parallel to the river to account for the gradient in soil texture. Each species-richness level was represented in equal numbers across the four blocks. Sowing density amounted to 1000 viable seeds per m^2^ where constant total density was achieved by reducing sowing densities per species proportionally to the number of species in the mixture (for details see [20]).

**Figure 1:**
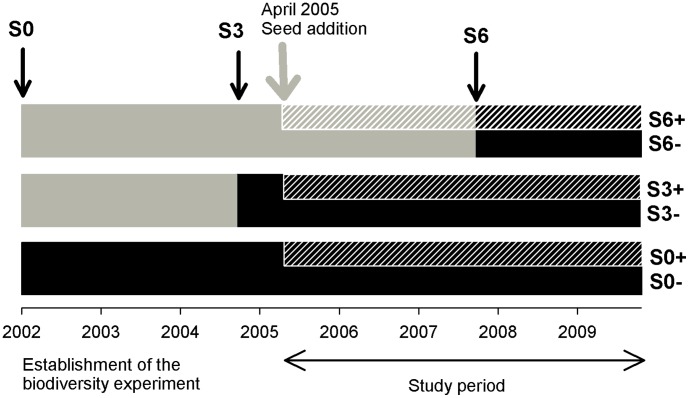
Summary of studied colonization period × seed addition treatments across initial species-richness levels. Vertical arrows indicate the time, when weeding was stopped in different treatments: (S0−)  =  never weeded, no seed addition, (S0+)  =  never weeded, seed addition (2005), (S3−)  =  cessation of weeding after three years (2004), no seed addition, (S3+)  =  cessation of weeding after three years (2004), seed addition (2005), (S6−)  =  cessation of weeding after six years (2007), no seed addition, and (S6+)  =  cessation of weeding after six years (2007), seed addition (2005).

Within each large plot of the biodiversity experiment, three pairs of 2.00×2.25 m subplots were established separated by 0.3 m distance from each other and at 0.5 m distance from the plot margin. One pair of these subplots was never weeded since the establishment of the experiment in 2002, while all species not included in the initially established species combinations were removed in the other subplots twice per year. Three years after sowing, one pair of the regularly weeded subplots was chosen randomly to stop weeding, while weeding was continued in the other subplot-pair. At the same time, one subplot of each weeding treatment was selected at random for seed addition. A mixture with seeds of all 60 species of the experimental pool at equal proportions was assembled for the seed addition treatment. Subplots were sown with a density of 1000 germinable seeds per m^2^ in April 2005 (for details see [21]). All non-experimental species were removed in the seed addition subplot of the continuous-weeding treatment during two yearly weeding campaigns (early April, July). Six years after establishment of the biodiversity experiment, weeding was also stopped in the subplot-pair with continuous weeding resulting in the following assembly-history treatments (Fig. 1):

(S6−) cessation of weeding after six years (2007), no seed addition,
(S6+) cessation of weeding after six years (2007), seed addition (2005),
(S3−) cessation of weeding after three years (2004), no seed addition,
(S3+) cessation of weeding after three years (2004), seed addition (2005),
(S0−) never weeded, no seed addition, and
(S0+) never weeded, seed addition (2005).

Plots were mown twice per year (June, September) and the mown plant material was removed.

### Data collection

Species richness, species identities and visually estimated species cover using a decimal scale [25] were recorded twice per year shortly before mowing in May and August from 2005 to 2009. In addition, species cover of colonizing species was estimated in all subplots in early April (start of the growing season) and July (re-growth after mowing) to get information on the cover of colonizing species, which were weeded in some treatments until 2007 (S6−, S6+) and to obtain reliable data on short-living spring or annual plants not belonging to the experimental species pool in other treatments. All species inventories per subplot (including the removed species in S6− and S6+) were summarized per year using the maximum observed cover.

A trait matrix comprising species mean traits related to spatial and temporal niche dimensions and to strategies of regeneration was compiled for all observed species ( = 236 species in total) based on information in the floristic literature [26], original literature (see Text S1) and databases (LEDA [27]; BiolFlor [28]). Additional data on specific leaf area were collected in 2009 for missing species at the field site or in surrounding habitats. Trait information was available for >90% of all species for every trait (Table 1).

**Table 1:** Overview of plant traits used in analyses of functional trait diversity and community-weighted mean traits.

Trait	Type of variable	Availability
Growth height	continuous (cm)	99%
Specific leaf area	continuous (mm^2^_leaf_ mg^−1^_leaf_)	95%
Growth form	ordinal (1 = rosulate; 2 = semirosulate; 3 = without basal leaf rosette)	100%
Seasonality of foliage	ordinal (1 = summergreen; 2 = overwintering green; 3 = evergreen)	100%
Life cycle	ordinal (1 = annual; 2 = biennial or monocarpic perennial; 3 = perennial)	100%
Seed mass	continuous (mg)	90%
Start of flowering period	ordinal (1 = before May; 2 = May; 3 = June; 4 = July)	98%
Duration of flowering period	ordinal (1 = two months or less; 2 = three months; 3 = four months; 4 = more than 4 months)	98%
Symbiotic N_2_ fixation	binary (0 = non, 1 = yes)	100%
Type of reproduction	ordinal (1 = by seed; 2 = mostly by seed, rarely vegetative; 3 = by seed and vegetative; 4 = mostly vegetative)	98%

### Data analyses

Bray-Curtis dissimilarities [29] using cover abundances per species derived from repeated inventories (see above) were calculated for each year (1) across communities initially established with 1 to 16 species (plots of the biodiversity experiment) for each treatment combination (subplots varying in seed addition × colonization period; S0+, S0−, S3+, S3−, S6+, S6−), and (2) within communities across treatment combinations (between subplots within the large plots). Similarly, Bray-Curtis dissimilarities were derived from community-weighted means of trait values (CWM) (Table 1) to test for dissimilarities in functional trait composition. CWMs were derived for each trait as the average of trait values weighted by the proportional abundance of species with the respective trait value [30]. CWMs were scaled to values between 0 and 1 before calculating Bray-Curtis dissimilarities. Because Bray-Curtis dissimilarity is based on pair-wise comparisons, all possible combinations were computed and averaged to get a mean value for each subplot.

Measures of functional trait diversity quantify the distribution of species in a multidimensional space whose axes are defined by functional characteristics. Three components of functional trait diversity were calculated separately: functional richness (FRic), functional evenness (FEve) and functional divergence (FDiv) [22], which have been identified to be more sensitive to community assembly rules than species richness [31]. FRic represents the amount of multidimensional trait space spanned out by a community and is quantified as the smallest convex set ( =  minimum convex hull) enclosing the volume of the *n*-dimensional trait space occupied by the species in a community [32]. FEve describes how regular the functional trait space is filled by species, weighted by their abundances. The calculation was based on the minimum spanning tree and represents the sum of branch length of all points contained in the *n*-dimensional trait space [22], [33]. FDiv estimates how abundance-weighted species diverge from the centre of gravity in the volume of the *n*-dimensional trait space [22]. Analyses of functional trait composition were based on ten functional traits (Table 1). Prior to analyses seed mass was log-transformed to reduce the influence of species with large trait values. A Gower dissimilarity matrix [34] was calculated to account for different types of trait data (numeric, ordinal, binary) and missing trait values [35]. Axes derived from a principal coordinate analysis (PCoA) and corrected by a method described by Cailliez [36] served to calculate the different indices of functional trait diversity [35]. To evaluate whether species richness and functional trait diversity converged through time, the coefficient of variation (CV) of these variables was computed across communities and within communities across treatment combinations for each year.

Linear mixed-effects models were applied to test for effects of the experimental factors on the response variables measured in the 5-year study period in the split-split-plot colonization experiment. Block and plot identity, subplot pair (S0, S3, S6) and subplot were treated as random factors in a nested sequence. Although the factors species richness and number of functional groups are nearly orthogonal in the Jena Experiment, the design of the experiment is not completely balanced because it is not possible to vary species richness and functional group number completely independently. Starting from a constant null model the fitting sequence of fixed terms followed the a-priori hypotheses of the biodiversity experiment starting with the number of initially established species (log-linear term), the number of functional groups (linear term), factors for the split-plot variables colonization period (with three factor levels) and seed addition (with two factor levels) and their interactions with the main experimental factors. Finally, a term for the different study years (as linear term to focus on directed temporal changes) and its interaction with the previously mentioned experimental factors was entered. The maximum likelihood method was used and likelihood ratio tests were applied to assess the statistical significance of model improvement. The statistical software R (Version 2.11.2, http://www.r-project.org) including the packages *FD* [37] and *nlme* [38] and PC-ORD 4.25 [39] were used for calculations and statistical analyses.

## Results

### Realized species richness

**Figure 2:**
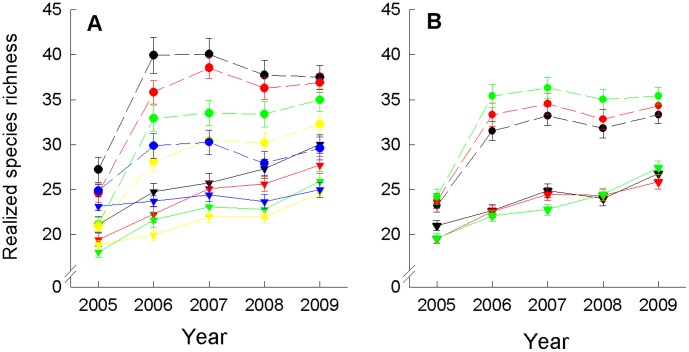
Realized species richness. Realized species richness as a function of time (A) as mean values (±1SE) per sown species-richness level across colonization periods, and (B) as mean values (±1SE) per colonization period across species-richness levels. For symbols see Figs. 3 and 4.

A total of 176 species in addition to the 60 experimental species were observed during the study period. An increased number of initially established species and functional groups allowed fewer species to colonize and thus resulted in lower realized species richness after colonization (Fig. 2, Table 2). The overall positive effect of seed addition on realized species richness was strongest when the initially established communities had a low number of species (significant interaction “SR × ADD”, Table 2, Fig. 2A). Although colonization period did not directly affect realized species richness, seed addition was most efficient when weeding of other than seed-added species was carried out for 3 years after seed addition (significant interaction “C × ADD”, Table 2, Fig. 2B). A continuous species gain through time was found in split-plots without seed addition, while seed-enriched split-plots did not assemble more species after an initial enrichment in the first two years after seed addition (Fig. 2). Coefficient of variation (CV) between communities reached peak values in the year after seed addition and declined in the subsequent years indicating an increasing convergence in realized species richness (Fig. S1A).

**Table 2:** Summary of mixed-effects model analyses for realized species richness, functional richness (FRic), functional evenness (FEve) and functional divergence (FDiv) based on a five-year study period from 2005–2009.

Source of variation	Species richness		FRic		FEve		FDiv	
	L	p	L	p	L	p	L	p
SR (log-linear)	8.53	0.004↓	11.75	0.001↓	41.27	<0.001↓	5.17	0.023↓
FG (linear)	5.71	0.017↓	4.78	0.029↓	1.26	0.261	0.52	0.470
Colonization period (C)	2.69	0.261	1.23	0.540	36.92	<0.001↓	1.45	0.485
SR (log-linear) × C	2.37	0.305	0.87	0.648	11.11	0.004	0.59	0.744
FG (linear) × C	0.34	0.845	1.46	0.481	0.21	0.903	0.74	0.690
Seed addition (ADD)	416.89	<0.001↑	396.65	<0.001↑	59.23	<0.001↑	15.99	<0.001↑
SR (log-linear) × ADD	54.55	<0.001	19.42	<0.001	9.41	0.002	3.31	0.069
FG (linear) × ADD	3.36	0.067	0.22	0.640	0.56	0.454	0.10	0.752
C × ADD	22.17	<0.001	16.06	<0.001	12.94	0.002	4.30	0.116
SR (log-linear) × C × ADD	9.98	0.007	3.44	0.179	9.77	0.008	8.46	0.015
FG (linear) × C × ADD	0.76	0.685	1.70	0.427	2.05	0.358	1.19	0.551
Year	583.18	<0.001↑	369.99	<0.001↑	175.34	<0.001↓	0.66	0.417
SR (log-linear) × Year	35.73	<0.001	51.56	<0.001	144.75	<0.001	0.70	0.403
FG (linear) × Year	0.84	0.359	6.52	0.011	2.75	0.097	4.39	0.036
C × Year	4.28	0.118	1.44	0.486	47.91	<0.001	0.11	0.948
ADD × Year	18.80	<0.001	39.37	<0.001	3.83	0.051	0.02	0.900

Models were fitted by stepwise inclusion of fixed effects. Listed are the results of likelihood ratio tests (L ratio) that were applied to assess model improvement and the statistical significance of the fixed effects (p values). Arrows indicate increase (↑) or decrease (↓) of the variables with species richness, functional group number, after seed addition, with increasing colonization period or with time; SR  =  initial species richness, FG  =  functional group number, ADD  =  seed addition in 2005, C  =  colonization period.

### Functional richness (FRic)

**Figure 3:**
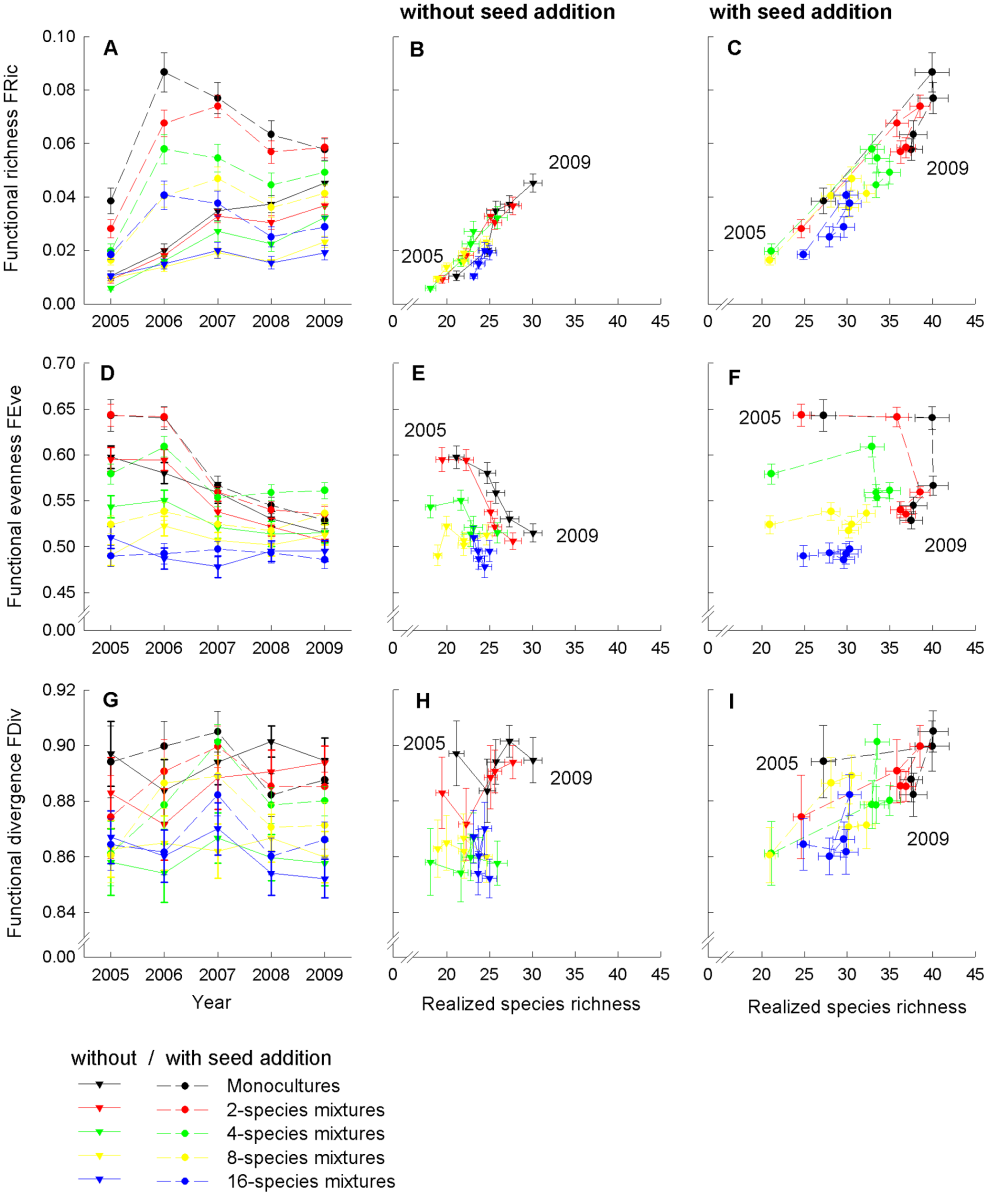
Functional richness (FRic), functional evenness (FEve) and functional divergence (FDiv) per initial species-richness level. FRic as a function of time (A), and plotted against realized species richness (B, C), FEve as a function of time (D), and plotted against realized species richness (E, F), and FDiv as a function of time (G), and plotted against realized species richness (H, I) as mean values (±1SE) per initial species-richness level across colonization periods. Treatments without seed addition are shown in (B, E, H), and treatments with seed addition are shown in (C, F, I).

**Figure 4:**
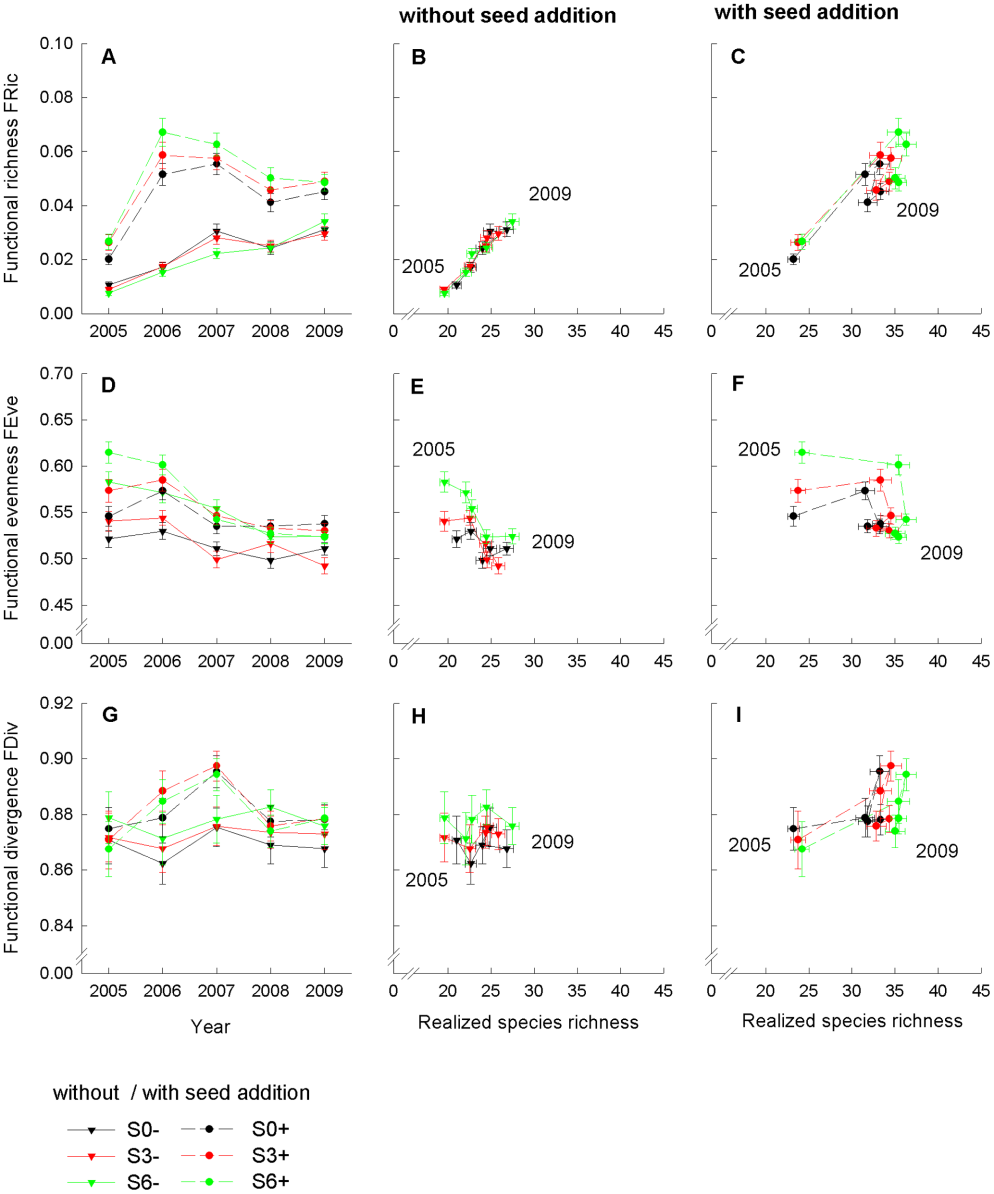
Functional richness (FRic), functional evenness (FEve) and functional divergence (FDiv) per colonization period. FRic as a function of time (A), and plotted against realized species richness (B, C), FEve as a function of time (D), and plotted against realized species richness (E, F), and FDiv as a function of time (G), and plotted against realized species richness (H, I) as mean values (±1SE) per colonization period across initial species-richness levels. Treatments without seed addition are shown in (B, E, H), and treatments with seed addition are shown in (C, F, I).

The higher resistance of communities with an increased number of initially established species and functional groups against colonization by new species resulted in a smaller increase in functional richness (FRic) after colonization of new species (Fig. 3A–C, Table 2). Seed addition had positive effects on FRic at all levels of initially sown species richness, but the efficiency of seed addition was reduced through the higher colonization resistance of more diverse initially established species combinations (significant interaction “SR × ADD”, Fig. 3A). Colonization period did not directly impact FRic, but the increment of FRic after seed addition was higher when weeding of species not added as seeds was continued for 3 years after seed addition (significant interaction “C × ADD”, Fig. 4A–C). FRic increased over the 5-year study period in subplots without seed addition and a low number of initially sown species (1 to 4 species), while FRic in subplots without seed addition and a higher number of initially sown species (8 and 16 species) remained unchanged at lower levels after an initial increment (Fig. 3B). Seed-enrichment resulted in peak values of FRic one year after seed addition, and declined again in the subsequent two years (Fig. 3C). Coefficient of variation (CV) decreased through time suggesting that FRic became more similar between communities (Fig. S1B).

### Functional evenness (FEve)

Realized functional evenness (FEve) after colonization declined with an increased number of initially established species (Fig. 3D–F, Table 2). A longer duration of weeding, i.e. a shorter colonization period, and seed addition increased FEve and had the largest positive effects in communities with a lower number of initially established species (significant interactions “SR × C” and “SR × ADD”; Figs. 3D–F, 4D–F) or when seeds were added in subplots where weeding of species was continued for 3 years after seed addition (significant interaction “C × ADD”). FEve decreased over the 5-year study period. Starting at higher levels, the loss of FEve was larger, when initially a lower number of species was established (Fig. 3E–F), as well as in split-plots with a longer continuation of weeding (Fig. 4E–F). FEve converged between communities with different initial species richness (decreasing CV) over the 5-year study period, but did not become more similar between treatments with different assembly history (Fig. S1C).

### Functional divergence (FDiv)

Realized functional divergence (FDiv) after colonization decreased with an increased number of initially established species (Fig. 3G–I, Table 2). Seed addition increased FDiv (Fig. 3G), while a longer continuation of weeding did not affect FDiv (Fig. 4G–I). Two years after seed addition, FDiv declined to similar values as in split-plots without seed addition (Table 2). Coefficient of variation (CV) in FDiv decreased over time between communities (Fig. S1D).

### Between-community dissimilarity in species and functional trait composition

**Figure 5:**
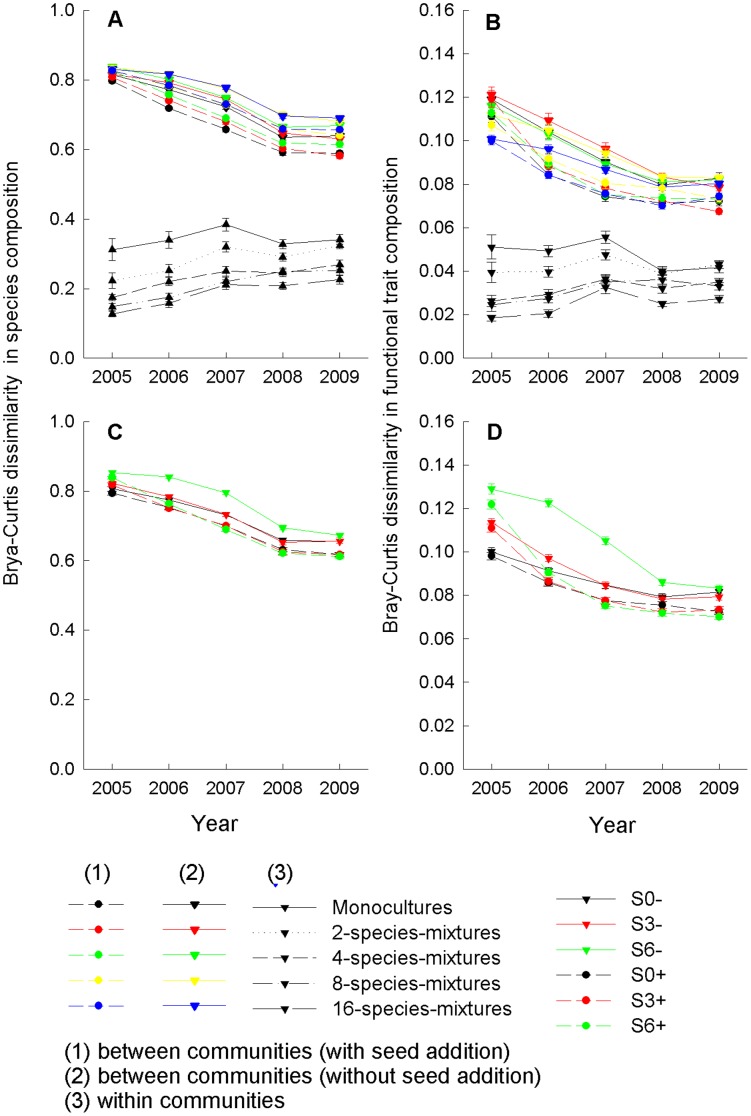
Between- and within-community dissimilarities in species and functional trait composition. Dissimilarities based on species composition (A, C), and functional trait composition (B, D) as a function of time. Between-community dissimilarities were calculated as mean pair-wise Bray-Curtis distances between all communities per colonization period × seed addition treatment, and within-community dissimilarities between split-plots with different assembly histories were calculated as mean pair-wise Bray-Curtis distances between colonization period × seed addition treatments per community. Mean values (±1SE) per initial species-richness level across colonization period × seed addition treatments (A, B), and mean values (±1SE) per colonization period × seed addition treatment across sown species-richness levels (C, D) are shown.

Average between-community dissimilarity in species composition was highest for communities initially established with a large number of species. Average between-community dissimilarity in functional trait composition did not depend on the initially established plant diversity (Table 3). Between-community dissimilarity in species and functional trait composition decreased through time (Fig. 5, Table 3). A lower number of initially established species, seed addition and earlier cessation of weeding (i.e. a longer colonization period) accelerated between-community convergence in functional trait composition and to a lesser extent in species composition. While between-community dissimilarity in functional trait composition decreased by 29.8% (±0.7 SE) during the study period of 5 years, between-community dissimilarity in species composition decreased by 22.2% (±0.3 SE).

**Table 3:** Summary of mixed-effects model analyses for dissimilarity (Bray-Curtis distances) based on species composition and functional trait composition: (A) between-community dissimilarity per colonization period × seed addition treatment, (B) within-community dissimilarity across colonization period × seed addition treatments.

Source of variation	Species composition			Functional trait composition		
(A) Between-community dissimilarity						
	L	p		L	p	
SR (log-linear)	25.17	<0.001	↑	0.46	0.496	
FG (linear)	0.29	0.591		0.28	0.597	
Colonization period (C)	44.00	<0.001	↓	97.20	<0.001	↓
SR (log-linear) × C	0.81	0.669		0.92	0.632	
FG (linear) × C	1.06	0.587		0.52	0.770	
Seed addition (ADD)	139.37	<0.001	↓	210.33	<0.001	↓
SR (log-linear) × ADD	2.68	0.102		5.37	0.020	
FG (linear) × ADD	0.65	0.419		0.78	0.378	
C × ADD	31.73	<0.001		63.48	<0.001	
SR (log-linear) × C × ADD	0.06	0.970		1.37	0.504	
FG (linear) × C × ADD	0.08	0.961		0.02	0.991	
Year	3595.11	<0.001	↓	1660.62	<0.001	↓
SR (log-linear) × Year	44.56	<0.001		45.86	<0.001	
FG (linear) × Year	0.08	0.781		0.66	0.418	
C × Year	68.84	<0.001		189.82	<0.001	
ADD × Year	49.15	<0.001		11.76	0.001	
(B) Within-community dissimilarity						
	L	p		L	p	
SR (log-linear)	77.34	<0.001	↓	85.19	<0.001	↓
FG (linear)	8.66	0.003	↓	8.53	0.004	↓
Year	104.39	<0.001	↑	22.20	<0.001	↑
SR (log-linear) × Year	12.11	0.001		26.53	<0.001	
FG (linear) × Year	0.53	0.468		0.53	0.466	

Models were fitted by stepwise inclusion of fixed effects. Listed are the results of likelihood ratio tests (L ratio) that were applied to assess model improvement and the statistical significance of the fixed effects (p values). Arrows indicate increase (↑) or decrease (↓) of the variables with species richness, functional group number, after seed addition, with increasing colonization period or with time; SR  =  initial species richness, FG  =  functional group number, ADD  =  seed addition in 2005, C  =  colonization period.

### Dissimilarity in species and functional trait composition caused by assembly-history treatments

The average within-plot dissimilarities (between different seed addition × colonization period combinations) in species and functional trait composition were smaller than between communities (Fig. 5A–B). Within-community dissimilarity in species and functional trait composition decreased with a higher number of initially established species and functional groups (Table 3). Species and functional trait composition in split-plots with different colonization period × seed addition treatments diverged in the first 3 years after seed addition. Divergence did not increase further in later years, and functional trait composition in communities initially established as monocultures or with two species even turned to convergence.

## Discussion

In spite of sustained efforts to understand patterns of community assembly, the importance of different biotic processes is poorly disentangled so far. We used the opportunity to study biotic processes of community assembly under relatively homogeneous environmental conditions in split-plots of a large grassland biodiversity experiment opened for colonization by new species and removed dispersal limitation through a seed addition treatment [21].

### Species richness and functional trait diversity

Species gain and increase in functional richness (FRic) after the opening of experimental communities for colonization by new species was reduced by a higher number of initially established species and a longer colonization period (shorter duration of weeding). The negative effects of a higher initially sown plant diversity on the spontaneous colonization of new species may be attributable to the lower stability of low-diverse communities even in the absence of weeding [40], while disturbance through weeding and the removal of competitors in communities with a shorter colonization period may have accelerated the colonization success of new species. The long-term effects of higher initial plant diversity in reducing the number of colonizer species are in line with previous short-term studies in the Jena Experiment and other biodiversity experiments (e.g. [41]–[43]). In our experiment, communities established with a lower number of species even exceeded realized species richness of communities established with a higher number of species after communities were opened for colonization (Fig. 2A). Seed addition led to a higher species gain and FRic increase than without seed addition, and the efficiency of seed addition was increased, when weeding was continued for a longer period. Higher levels of realized species richness after seed addition are commonly found because plant communities are often under-saturated with species due to a limited species pool or dispersal constraints [44], [45]. Although none of the species added as seed failed to establish completely after seed addition [21] and single communities accumulated indeed up to 60 species one or two years after seed addition, average species richness in the seed addition treatments declined again and did not exceed 35 species in the last year of the study (Fig. 2A). This upper limit of realized species richness is close to the realized species number in plots initially established with all 60 experimental species in the Jena Experiment (unpubl. data) and indicates that the seed addition led to forced colonization with a short-term over-saturation with species followed by species loss.

Functional richness (FRic), which quantifies the volume of functional space filled by a community, increased in parallel to species gain in sub-plots without seed addition, but the increment of FRic was flattened in subplots with a higher number of initially sown species (Fig. 3B). The concurrent increase in species richness and FRic indicated that newly colonizing species were functionally different from residents, which is in line with the view that complementary resource requirements of resident and colonizer species select for a maximization of functional richness (indicating limiting similarity). In contrast, FRic reached maximum values in the year after the seed addition in sub-plots with seed addition (Fig. 3C), followed by maximum values of functional divergence (FDiv) in the third year (Fig. 3I) before both measures of functional trait diversity declined more strongly than realized species richness in subsequent years. Functional evenness was also higher after seed addition, but decreased continuously through time (Fig. 3F). Decreasing FEve in parallel to species gain (Fig. 3E) and a minor trend in FDiv (Fig. 3H) through time, however, occurred in subplots without seed addition. FDiv describes how abundance is spread along functional trait axes, where large values of FDiv are expected to indicate a high degree of niche differentiation [19]. Our analysis of functional trait diversity was based on multiple traits. Although different functional traits may be associated with different processes during community assembly and relate to different niche axes [46], [47], plants have to balance different functions which is often reflected in fundamental functional trade-offs. Probably, the short-term increase in FDiv and maximum levels of FRic after seed addition were due to the advantage of species with traits associated with rapid establishment followed by weaker competitor exclusion. Weaker competitor exclusion (related to equalizing fitness processes) in grasslands at high productivity levels is discussed as a biotic process resulting in niche reduction in favour of perennial species with higher ability for resource competition [6], [48], [49]. Decreasing FEve may be either due to abundances less evenly distributed among species or to less regular functional distances among species [22]. The decline in FEve through time as well as a reduced FEve in subplots with a longer colonization period (Fig. 4D–F) indicated again that equalizing fitness processes favoured species with more similar trait combinations during community assembly.

### Convergence in species and functional trait composition

Previous studies have reported that experimental communities initially sown with different plant diversity and opened to colonization converged in species richness and functional or phylogenetic composition while keeping different species composition [13], [50]. Our results based on a 5-year study period starting 4 years after establishing communities with different plant diversity also showed stronger between-community convergence in functional trait composition than in species composition. In subplots with a different assembly history, however, we found a divergence in communities initially established with higher plant diversity (4 and more species, Fig. 5B), while communities initially established as monocultures or 2-species mixtures maintained their initial convergent state. Probably, this divergence at higher species-richness levels was due to the stochasticity in colonization processes and the persistence of priority effects, which may cause high variability in community structure among similar sites [51].

It becomes increasingly apparent that multiple processes are involved in community assembly. A number of recent studies have shown that patterns of functional trait composition depend on environmental conditions and the traits considered [46], [48], [49], [52]. Our study based on multiple traits provided evidence that contrasting biotic processes govern shifts of functional community composition in experimental grasslands with relatively homogeneous environmental conditions. Limiting similarity played a larger role during early phases of colonization. Colonization of new species went in parallel with an increase in FRic indicating that the newly colonizing species were functionally different from residents (hypothesis 1). Increasing FRic was succeeded by a decline in FEve suggesting weaker competitor exclusion in the longer run (hypothesis 2). These processes were accelerated when seed limitation was removed through seed addition resulting in peak values of FRic and larger values of FEve, which declined again in subsequent years (hypothesis 3). Nevertheless, FRic and FEve remained at higher levels in subplots with seed addition compared to those without seed addition during the 5-year study period, indicating that the removal of seed limitation improved the chance for assembling communities with a higher number of species with traits advantageous in competition. However, in addition to the series of assembly processes related to limiting similarity and weaker competitor exclusion, our study has clearly shown that historical contingency in assembly processes due to priority effects may be traceable over longer time (hypothesis 4). First, species gain and increase in FRic were considerably reduced when plant communities were initially established with greater diversity such that, realized species richness and FRic of initially low-diverse communities were even greater than in initially high-diverse communities after opening for colonization. Second, a longer duration of the colonization period decreased the accelerated species gain and the increase in FRic in initially low-diverse communities and maintained FEve at lower levels. In addition, the prominent role of priority effects becomes visible by the lagged convergence of species composition relative to functional trait composition of communities initially established with different species compositions (hypothesis 5).

## Supporting Information

Figure S1 Between- and within-community coefficients of variation of realized species richness, FRic, FEve and FDiv.
(DOC)

Text S1 Reference list for data on specific leaf area of particular plant species included in the matrix of functional traits.
(DOC)
