# Supplementary material for: Different Assembly Processes Drive Shifts in Species and Functional Composition in Experimental Grasslands Varying in Sown Diversity and Community History
Source: PLoS One. 2014 Jul 16;9(7):e101928. doi: 10.1371/journal.pone.0101928 (PMC4100744; doi:10.1371/journal.pone.0101928)
Supplement: Figure S1 — Between- and within-community coefficients of variation of realized species richness, FRic, FEve and FDiv. (DOC) [file pone.0101928.s001.doc]

**Figure S1. Between- and within-community coefficients of variation of realized species richness, FRic, FEve and FDiv.** Coefficient of variation (CV) of (A) realized species richness, (B) functional richness (FRic), (C) functional evenness (FEve), and (D) functional divergence (FDiv) as a function of time between communities per colonization period × seed addition treatment, and within communities across colonization period × seed addition treatments. Shown are means (±1SE). Mixed-effect models were used to test for a decline in CV through time (= convergence).
