# Supplementary material for: Different Assembly Processes Drive Shifts in Species and Functional Composition in Experimental Grasslands Varying in Sown Diversity and Community History
Source: PLoS One. 2014 Jul 16;9(7):e101928. doi: 10.1371/journal.pone.0101928 (PMC4100744; doi:10.1371/journal.pone.0101928)
Supplement: Text S1 — Reference list for data on specific leaf area of particular plant species included in the matrix of functional traits. (DOC) [file pone.0101928.s002.doc]

**Text S1. Reference list for data on specific leaf area of particular plant species included in the matrix of functional traits.**

Cornelissen JHC, Castro Diez P, Hunt R (1996) Seedling growth, allocation and leaf attributes in a wide range of woody plant species and types. J Ecol 84: 755–765.

Fanelli G, DeLillis M (2004) Relative growth rate and hemerobiotic state in the assessment of disturbance gradients. Appl Veg Sci 7: 133-140.

Neugebauer KR (2003) Auswirkungen der extensiven Freilandhaltung von Schweinen auf Gefäßpflanzen in Grünlandökosystemen. Dissertation, Universität Regensburg.

Schädler M (2001) Einfluß phytophager Insekten auf die Struktur und Dynamik einer Ackerbrache. Dissertation, Universität Halle-Wittenberg.

Storkey J (2006) A functional group approach to the management of UK arable weeds to support biological diversity. Weed Res 46: 513-522.

Tharakan PJ, Volk TA, Nowak CA, Abrahamson LP (2005) Morphological traits of 30 willow clones and their relationship to biomass production. Can J For Res 35: 421-431.

Vile D, Shipley B, Garnier E (2006) A structural equation model to integrate changes in functional strategies during old-field succession. Ecology 87: 540-517.
